# Combination of the natural product capsaicin and docetaxel synergistically kills human prostate cancer cells through the metabolic regulator AMP-activated kinase

**DOI:** 10.1186/s12935-019-0769-2

**Published:** 2019-03-08

**Authors:** Belén G. Sánchez, Alicia Bort, Pedro A. Mateos-Gómez, Nieves Rodríguez-Henche, Inés Díaz-Laviada

**Affiliations:** 1 Department of Systems Biology, Biochemistry and Molecular Biology Unit, School of Medicine and Health Sciences, Alcala University, Alcalá de Henares, Ctra A-2 Km 32., 28871 Madrid, Spain; 2 Chemical Research Institute “Andrés M. del Río” (IQAR), Alcalá University, Alcalá de Henares, 28871 Madrid, Spain

**Keywords:** Docetaxel, Capsaicin, AMPK, PC3 cells, LNCaP cells, Prostate cancer

## Abstract

**Background:**

Current chemotherapy for castration-resistant prostate cancer is established on taxane-based compounds like docetaxel. However, eventually, the development of toxic side effects and resistance limits the therapeutic benefit being the major concern in the treatment of prostate cancer. Combination therapies in many cases, enhance drug efficacy and delay the appearance of undesired effects, representing an important option for the treatment of castration-resistant prostate cancer. In this study, we tested the efficacy of the combination of docetaxel and capsaicin, the pungent ingredient of hot chili peppers, on prostate cancer cells proliferation.

**Methods:**

Prostate cancer LNCaP and PC3 cell lines were used in this study. Levels of total and phosphorylated forms of Akt, mTOR, S6, LKB1, AMPK and ACC were determined by Western blot. AMPK, LKB1 and Akt knock down was performed by siRNA. PTEN was overexpressed by transient transfection with plasmids. Xenograft prostate tumors were induced in nude mice and treatments (docetaxel and capsaicin) were administered intraperitoneally. Statistical analyses were performed with GraphPad software. Combination index was calculated with Compusyn software.

**Results:**

Docetaxel and capsaicin synergistically inhibited the growth of LNCaP and PC3 cells, with a combination index lower than 1 for most of the combinations tested. Co-treatment with docetaxel and capsaicin notably decreased Akt and its downstream targets mTOR and S6 phosphorylation. Overexpression of PTEN phosphatase abrogated the synergistic antiproliferative effect of docetaxel and capsaicin. The combined treatment also increased the phosphorylation of AMP-activated kinase (AMPK) and the phosphorylation of its substrate ACC. In addition, pharmacological inhibition of AMPK with dorsomorphin (compound C) as well as knock down by siRNA of AMPK or its upstream kinase LKB1, abolished the synergy of docetaxel and capsaicin. Mechanistically, we showed that the synergistic anti-proliferative effect may be attributed to two independent effects: Inhibition of the PI3K/Akt/mTOR signaling pathway by one side, and AMPK activation by the other. In vivo experiments confirmed the synergistic effects of docetaxel and capsaicin in reducing the tumor growth of PC3 cells.

**Conclusion:**

Combination of docetaxel and capsaicin represents a therapeutically relevant approach for the treatment of Prostate Cancer.

**Electronic supplementary material:**

The online version of this article (10.1186/s12935-019-0769-2) contains supplementary material, which is available to authorized users.

## Background

Prostate cancer (PCa) is the most prevalent malignancy in men worldwide, and the second leading cause of cancer related deaths [1, 2]. Environmental factors such as hypercaloric diets, sedentary life, increasing life expectancy and modified diagnostic techniques contribute to the increase in prostate cancer incidence. For locally advanced and metastatic cancers androgen deprivation therapy is the standard of care. Despite initial disease regression, most men eventually progress to castration-resistant prostate cancer (CRPC) with no response to hormonal therapy and a lethal outcome. Currently, docetaxel is the first-line chemotherapeutic agent available to patients with this lethal form of the disease, but the survival of patients remains limited by the occurrence of dose-dependent adverse effects and acquired resistance. Mechanisms underpinning resistance development include overexpression of multidrug efflux pumps, mutation of β-tubulin, and activation of signaling proteins as MAPK or Akt [3]. Docetaxel resistance is a clinical problem since it is the main therapy for CRPC. Moreover, newer chemotherapeutic drugs developed to treat docetaxel resistant patients carry significant hematological toxicities [3]. Therefore, approaches to improve taxane-based chemotherapy are urgently required [4]. Thus, it is of highly clinical significance to identify agents that when combined with the current chemotherapeutic drugs allow to decrease the doses without reducing their effectiveness as well as to avoid and/or to overcome drug resistance. Therefore, combination therapy, a treatment modality that combines two or more therapeutic agents, is becoming a cornerstone of cancer therapy [5].

Over the past few years, many anti-cancer drugs have been identified from natural nutritional compounds. Capsaicin (CAP), the spicy ingredient of hot chili peppers, exhibit anti-neoplastic activity in many cancer cell lines as well as in vivo [6]. In addition, recent data indicate that CAP sensitizes cells to chemotherapeutic agents. For instance, the combination of CAP and camphothecin increases apoptosis in small cell lung cancer [7]. In cholangocarcinoma, CAP increases sensitivity to 5-fluorouracil and the mixture of both compounds inhibits tumor growth with greater efficacy than 5-fluorouracil alone [8]. In human prostate cancer cells CAP combined with brassinin enhances apoptotic and anti-metastatic effects [9]. We have shown that, in hepatocellular carcinoma cells, CAP increases the antiproliferative effects of sorafenib [10]. Yet, the mechanisms underlying the capsaicin-mediated inhibition of cell proliferation and drug sensitization are divers and poorly understood. Laboratory data supports the notion that dietary capsaicin has anti-obesity role by increasing energy expenditure, enhancing fat oxidation, decreasing adipogenesis and suppressing appetite [11]. Although a molecular mechanism has not been clarified, all these functions may be regulated by the AMP-activated kinase (AMPK).

The cellular metabolic sensor AMPK has emerged as a key therapeutic target for many cancers. Besides its role in energy homeostasis, AMPK blocks cell cycle, induces apoptosis, regulates autophagy and suppresses the anabolic processes required for rapid cell growth [12]. Moreover, pharmacological activation of AMPK by the antidiabetic drug metformin, has been demonstrated to sensitize cancer cells to cytotoxic therapy [13]. AMPK is a heterotrimeric protein consisting of a catalytic α subunit, and regulatory β and γ subunits. It is activated by AMP binding to the γ subunit as well as by phosphorylation of the Thr172 residue of α subunit mainly by LKB1 kinase although other upstream kinases have also been described [14].

In this study we evaluated the ability of CAP to inhibit prostate cancer cell proliferation. We found that CAP synergizes with docetaxel to potently block cell growth in vitro and tumor growth in vivo by a mechanism involving activation of AMPK.

## Materials and methods

### Materials

Capsaicin (CAP) and Ddocetaxel (DTX) were purchased to TOCRIS (Bristol, UK). dorsomorphin and STO-609 were purchased to Sigma (St. Louis, USA). Primary antibodies anti-pAMPKα1-thr172, pACC-ser79, pAkt-ser473, pmTOR, pS6, pLKB1 and the antibodies against the corresponding total forms were obtained from Cell Signaling Technology (Danvers, MA, USA). Peroxidase labeled secondary anti-mouse IgG was from Sigma (St. Louis, MO, USA) and anti-rabbit IgG was from Calbiochem (San Diego, USA).

### Cell culture

PC3 and LNCaP human prostate cancer cell lines were obtained from American Type Culture Collection (ATCC CRL-1435 and ATCC CRL-1740 respectively) (Rockville, MD, USA). Cells were routinely grown in RPMI 1640 medium supplemented with 100 IU/ml penicillin G sodium, 100 μg/ml streptomycin sulfate, 0.25 μg/ml amphotericin B (Invitrogen, Paisley, UK) and 10% fetal bovine serum. For treatment experiments, cells were plated and grown 48 h, the medium was then replaced with serum-free RPMI 1640 for 24 h and then incubated with different treatments for the indicated times. Cells were used at passages 4–20.

### Cell viability assay (MTT)

Cell viability was measured by MTT (3-(4,5-dimethylthiazol-2-yl)-2,5-diphenyltetrazolium bromide) Cell Proliferation assay (Sigma, St. Louis, MO, USA) 24 h after exposure to treatments. In brief, a total of 5000 cells/well were seeded into 12-well plate in a final volume of 1 ml. After treatments, 100 µl MTT solution (5 mg/ml in PBS) was added to the medium and cells were incubated at 37 °C for 4 h. Then, the supernatant was discarded and dimethyl sulfoxide was added to dissolve the formazan crystals. Treatments were carried out in triplicate. The optical density in each well was evaluated by measurement of absorbance at 490 and 650 nm using an iMark™ Absorbance Reader from Bio-Rad (Richmond, CA, USA).

### Western blot analysis

Cells were lysed in a lysis buffer (50 mM Tris pH 7.4, 0.8 M NaCl, 5 mM MgCl_2_, 0.1% Triton X-100) containing Protease Inhibitor and Phosphatase inhibitor Cocktail (Roche, Diagnostics; Mannheim, Germany), incubated on ice for 15 min and cleared by microcentrifugation. Protein concentrations were measured by BioRad™ protein assay kit (Richmond, CA, USA). Cell proteins extracts (20 μg) were separated by sodium dodecyl sulfate–polyacrylamide gel electrophoresis (SDS-PAGE) and then transferred onto a PVDF membrane. Thereafter, nonspecific binding was blocked with 5% of BSA in TTBS for 1 h at room temperature. Membranes were then incubated overnight at 4 °C with primary antibodies. After washing in TTBS, membranes were incubated with peroxidase-conjugated anti-mouse or anti-rabbit secondary antibodies (1:2000) for 2 h at room temperature. The immune complex was visualized with an ECL system (Cell Signaling Technology).

### siRNA transfections

Cells were transfected in 1 ml OptiMEM (Invitrogen, Carlsbad, CA, USA) containing 4 µg lipofectamine iMax (Invitrogen, Carlsbad, CA), with 100 nM AMPK specific siRNA duplexes (5′-CCCAUAUUAUUUGCGUGUAdTdT-3′ and 5′-UACACGCCAAAUAAUAUGGGdTdT-3′), LKB1 selective siRNA duplexes (5′-GUACUUCUGUCAGCUGAUUdTdT-3′ and 5′-AAUCAGCUGACAGAAGUACdTdT-3′) (Sigma, St. Louis, MO, USA), Akt selective siRNA duplexes (Cell Signaling Technology, Danvers, MA, USA) or control scrambled RNA (Invitrogen, Carlsbad, CA) for 72 h according to manufacturer’s protocols. At 72 h after transfection, the medium was removed and replaced for RPMI containing 10% fetal bovine serum. At dedicated time points after transfection, cells were used for MTT cell viability assays or Western blot.

### PTEN transfections

The plasmid encoding full-length human PTEN was provided by Jaewhan Song (Addgene plasmid # 78777; http://n2t.net/addgene:78777; RRID: Addgene_78777 [15], Addgene Watertown, MA, USA). PC3 cells were seeded in 6 or 12 well plates with complete medium and transfected with 4 μg of PTEN recombinant plasmid (pcDNA3-FLAG PTEN), using 5 μl of Lipofectamine 3000 (Thermofisher, Waltham, MA, USA) and OptiMEM. After 48 h of transfection, the medium was replaced by another without serum and the different treatments were administered. Subsequently, cell viability was assessed by MTT and protein expression was analyzed by Western blot using anti-FLAG antibodies (Flag M2 antibody, Sigma, Saint Louis, MO, USA).

### Anti-tumor activity in mouse tumor models

Four-week-old athymic nude-Foxn1 (nu/nu) mice were purchased from Envigo RMS (Barcelona, Spain) and housed in a laminar air-flow cabinet under pathogen-free conditions on a 12-h light/dark schedule at 21–23 °C and 40–60% humidity with access to food pellets and tap water ad libitum. 4 animals were housed by cage. G Power analysis was used to calculate sample size [16], according to our previous data and experience and considering two tails effect and a significance level of 5%. Prostate tumors were induced in athymic mice by subcutaneal injection of 5 × 10^6^ PC-3 cells or 5 × 10^6^ LNCaP cells, according to the experiment. When tumors reached 70 mm^3^ the mice were randomly divided into four experimental groups of 6 animals each, and the following treatments were started by daily i.p. injection: Vehicle (DMSO), 2 mg/kg capsaicin (CAP), 10 mg/kg docetaxel (DTX) or 2 mg/kg capsaicin + 10 mg/kg docetaxel (CAP + DTX). Tumor sizes were measured every day and calculated using the formula V (mm^3^) = 1/2(Length × Width^2^). At the end of the study, the mice were sacrificed by placing them in a CO_2_ gas-filled chamber, and the excised tumors were recovered and weighted.

### Combined drug analysis

Drug interaction was determined using the combination index (CI)-isobologram equation that allows quantitative determination of drug interactions, where CI < 1 implied synergism, CI = 1 additive, and CI > 1 implied antagonism [17, 18]. Compusyn^©^ version 1.0 software (ComboSyn, Inc. Paramus, NJ, USA) was used to generate the dose–response curves, dose–effect analysis, and CI-effect plot.

### Statistical analysis

The statistical analysis of the results was performed using a two-way ANOVA and Dunnett’s multiple comparisons test or Tukey’s multiple comparisons test. The results were reported as mean ± SEM or SD as indicated in figure caption, of at least three independent experiments Data were considered significant when p ≤ 0.05.

## Results

### Capsaicin and docetaxel synergistically inhibit prostate cancer cells growth

**Fig. 1:**
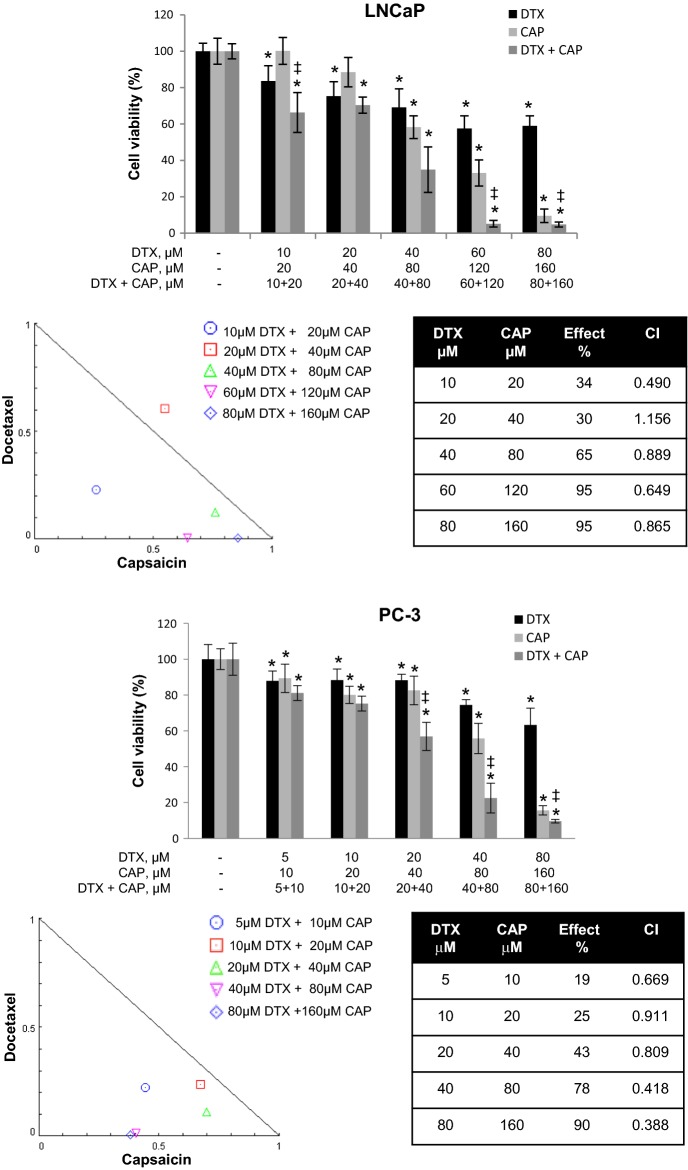
Synergistic antiproliferative effect of capsaicin and docetaxel on prostate tumor cells. LNCaP cells (upper panel) or PC3 cells (lower panel) were incubated with increasing doses of docetaxel (DTX), capsaicin (CAP) or the combination of both compounds for 24 h and cell viability was evaluated by MTT. Data represent the mean (n = 3) ± SD. *p < 0.0001 significant difference between treated and control cells by two-way ANOVA and Dunnett’s multiple comparisons test; ^‡^p < 0.0001 indicates significant interaction between DTX and CAP treatment. Data were analyzed with CompuSyn software to calculate the combination index (CI)

To assess the antiproliferative effect of docetaxel (DTX) and capsaicin (CAP), prostate cancer LNCaP and PC3 cells were incubated with increasing doses of DTX, CAP or the combination of both compounds and cell viability was determined by MTT. As shown in Fig. 1, both DTX and CAP singly reduced cell viability of LNCaP and PC3 cells. CAP alone dose-dependently inhibited cell viability from 40 µM in LNCaP cells and from 20 μM in PC3 cells. It is worthy to note that 50% inhibitory concentration (IC50) was much higher for DTX (300 μM) than for CAP (72 μM) in PC3 cells. When DTX and CAP were combined a dramatic reduction on cell viability was observed. We tested a constant CAP:DTX ratio (2:1) to create isobolograms using CompuSyn^©^ software and to calculate the combination index (CI). CI allows the quantification of synergism or antagonism for two drugs where CI of 1 indicates an additive effect, whereas a CI < 1 or CI > 1 indicates synergism or antagonism, respectively. Combination-index showed a potent synergy of cell killing at four of the five combinations used in LNCaP cells and at the five combinations used for PC3 cells. Likewise, isobologram for the combination of docetaxel and capsaicin showed that all combination data points in PC3 cells fall on the lower left, indicating synergism (Fig. 1). In LNCaP the synergic effect was evident in four combinations, but it was less strong that in PC3 cells. Then, for further experiments we chose the combination of DTX 40 μM + CAP 80 μM as it had a high synergistic effect but a moderate cytotoxic effect.

**Fig. 2:**
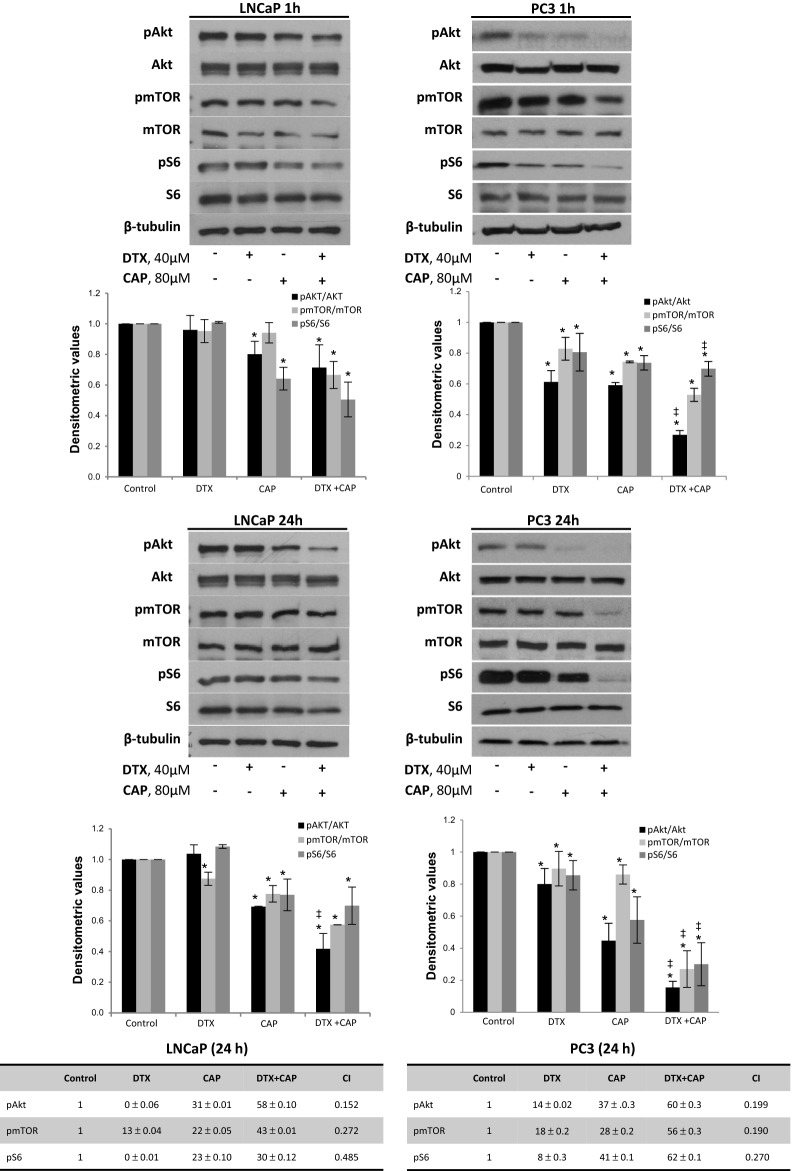
Combination of docetaxel and capsaicin effectively inhibits Akt/mTOR pathway. LNCaP or PC3 cells were incubated with 40 μM docetaxel (DTX), 80 μM capsaicin (CAP) or the combination of both during 1 h or 24 h and levels of phospho-Akt, phospho-mTOR, phospho-S6 and their corresponding total forms were determined by Western blot. β-Tubulin serves as a loading control. The densitometric analyses of bands represented as the mean ± SD of three different experiments are shown below the plots. *p < 0.05 significant difference between treated and control cells by two-way ANOVA and Dunnett’s multiple comparisons test; and ^‡^p < 0.05 indicate significant interaction between DTX and CAP treatment

Akt is a major component of the PI3K signaling pathway which is constitutively active in PC3 and LNCaP cells due to a PTEN deletion. Activation of the PI3K axe is involved in many cell functions that induce cell growth. To corroborate the antiproliferative effect of the combination of DTX and CAP, we evaluated the phosphorylation of Akt and its downstream effectors mTOR and S6. Results indicated that DTX induced a modest inhibition of Akt, mTOR and S6 phosphorylation only in PC3 cells and that CAP diminished Akt, mTOR and S6 phosphorylation both in PC3 and LNCaP cells at 1 h and 24 h (Fig. 2). However, co-treatment with DTX and CAP leaded to a higher inhibition of Akt phosphorylation in LNCaP cells and suppressed Akt phosphorylation in PC3 cells, both at 1 h and 24 h (Fig. 2). Likewise, the phosphorylation of mTOR and S6 were also significantly inhibited in the co-treated PC3 cells (Fig. 2). In LNCaP cells the combined treatment also caused inhibition of mTOR and S6 phosphorylation but the effect was weaker than that of PC3 cells even at 24 h, indicating that the combined treatment is more efficient in the castration resistant prostate PC3 cells (Fig. 2). ANOVA analysis revealed that the inhibitory effect produced by DTX + CAP on Akt, mTOR and S6 phosphorylation was different from that produced by DTX or CAP given singly. Moreover, the inhibitory effect on Akt, mTOR and S6 at 24 h in the co-treated cells was higher than the sum of DTX and CAP individual effects and combination index was lesser than 1, indicating synergism (Fig. 2). Those results suggest that CAP and DTX synergistically block the PI3K route which could underly the antiproliferative effect of DTX and CAP combination on prostate cancer cells.

**Fig. 3:**
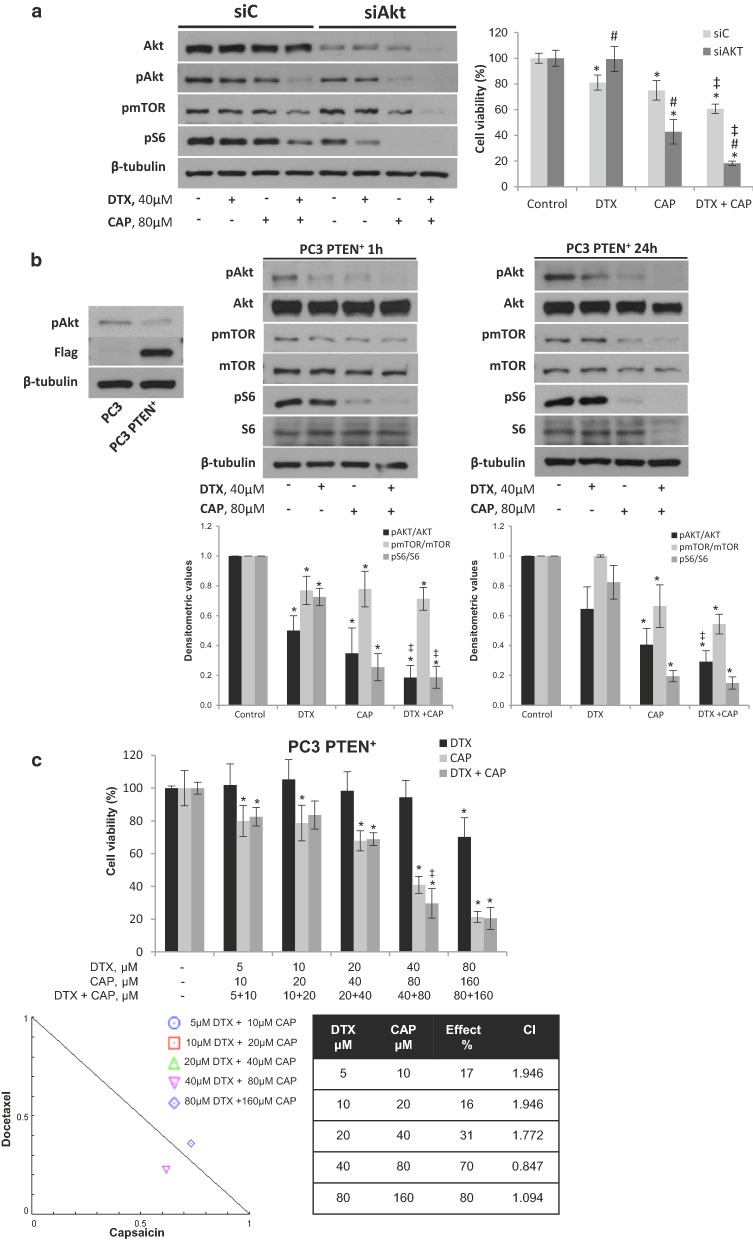
Targetting PI3K/Akt pathway hampers the inhibitory effect of docetaxel and abrogates synergy. **a** Akt was knocked down in PC3 cells by transfection with selective siRNA. Levels of phospho-Akt, phospho-mTOR, phospho-S6 and their corresponding total forms determined by Western blot is shown on the left. Cell viability by MTT is shown on the right. **b** PTEN phosphatase was overexpressed in PC3 by transient transfection with a plasmid containing full-length human PTEN and Flag and levels of phospho-Akt, phospho-mTOR, phospho-S6 and their corresponding total forms were determined by Western blot. The densitometric analyses of bands is shown below. **c** Cell viability and isobologram of PC3 cells transfected with PTEN plasmid and treated with DTX, CAP or both. *p < 0.05 significant difference between treated and control cells by two-way ANOVA and Dunnett’s multiple comparisons test; ^‡^p < 0.05 indicates significant interaction between DTX and CAP treatment; ^#^p < 0.05 significant different between siAKT and siC by two-way ANOVA and Sidak’s multiple comparisons test

To further corroborate this notion, we knocked down Akt by siRNA and determined cell viability by MTT. As shown in Fig. 3a, Akt pathway blockage abolished DTX inhibitory effect while enhanced CAP inhibitory effect on PC3 cell viability suggesting that DTX needs Akt to exert its antiproliferative effect. To additionally explore this pathway, we overexpressed the phosphatase PTEN in PC3 cells by transient transfection with a plasmid containing a flag tagged human PTEN. As shown in Fig. 3b, overexpression of PTEN in PC3 cells did not prevent the inhibitory effect of CAP and DTX + CAP on Akt, mTOR and S6 phosphorylation but hindered the inhibitory effect on cell viability (Fig. 3c). Moreover, the synergistic antiproliferative effect of the combined treatment was lost at four of the five combinations tested (Fig. 3c). These findings indicate that activation of the PI3K/Akt pathway is involved in the synergistic antiproliferative effect elicited by the combination of DTX and CAP in the castration resistant prostate PC3 cells.

### AMPK activation is involved in the antiproliferative effect of CAP + DTX

**Fig. 4:**
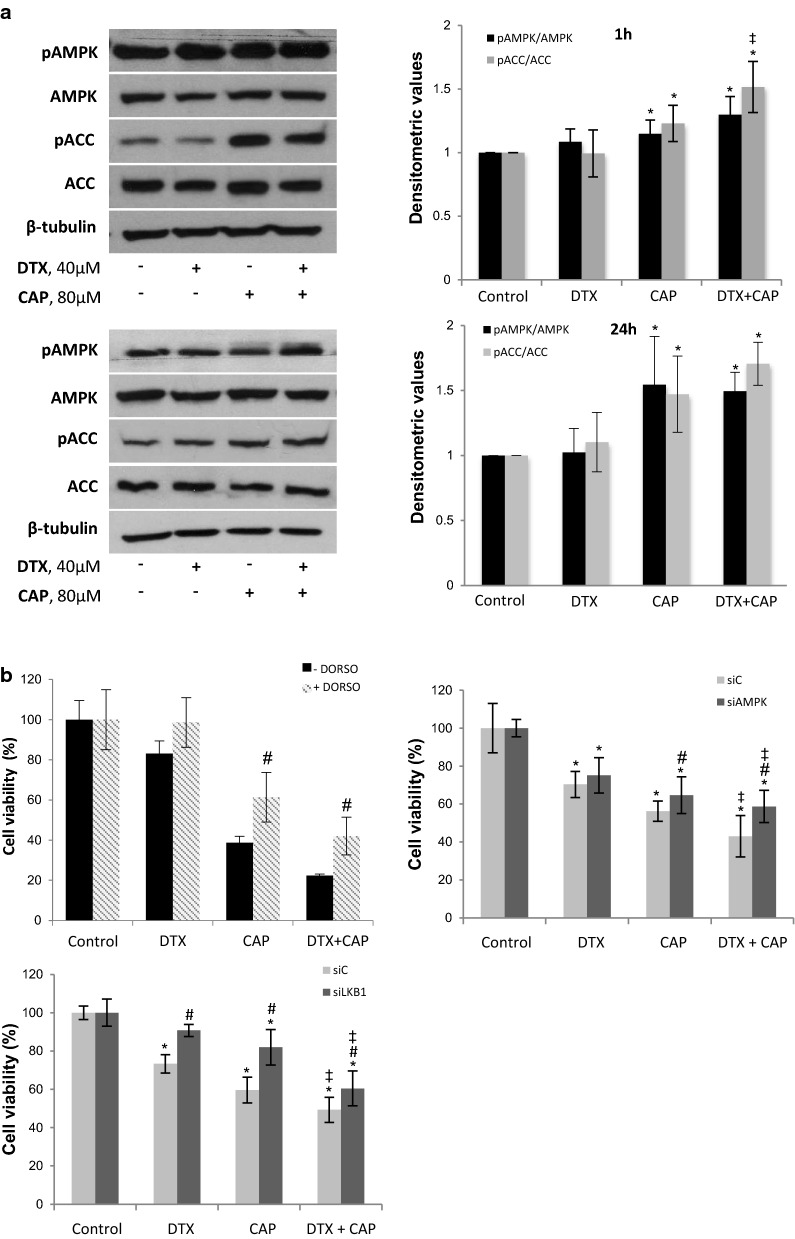
Combination of docetaxel and capsaicin activates AMPK. **a** PC3 cells were incubated with 40 μM docetaxel (DTX), 80 μM capsaicin (CAP) or the combination of both during 1 h or 24 h and levels of phospho-AMPK, phospho-ACC and their corresponding total forms were determined by Western blot. β-Tubulin serves as a loading control. The densitometric analyses of bands is shown on the right. **b** Effect of the AMPK inhibitor dorsomorphin (DORSO, 5 μM), of AMPK silencing by siRNA or LKB1 silencing by siRNA on cell viability. Data are presented as the mean ± SD of three different experiments. *p < 0.05 significant difference between treated and control cells; ^#^p < 0.05 significant difference between non-silenced and silenced cells by two-way ANOVA and Dunnett’s multiple comparisons test; ^‡^p < 0.05 indicates a significant interaction between DTX and CAP treatment

Clinical data indicate that AMPK activation by Metformin, an antidiabetic biguanide, improve the survival of castration-resistant prostate cancer patients through sensitization to chemotherapy [19]. To test whether AMPK activation was associated with the synergistic effect of CAP and DTX, we determined levels of phosphorylated AMPK in the Thr172 of the α catalytic subunit and phosphorylation of its well-known substrate Acetyl CoA carboxylase (ACC). As shown in Fig. 4a, when PC3 cells were incubated with CAP an increase in pAMPK as well as of pACC was observed, both at 1 h and 24 h. The co-treatment with DTX and CAP produced a higher increase in AMPK and ACC phosphorylation indicating a strong AMPK activation. We next determined the influence of the AMPK activation on cell viability. To that end, we use the AMPK inhibitor Dorsomorphin (DORSO, also known as compound C) or we knocked down AMPK or its upstream kinase LKB1 with selective siRNA. As shown in Fig. 4b, AMPK inhibitor significantly prevented the cell viability decrease in co-treated cells (Fig. 4b). Moreover, AMPK knockdown as well as LKB1 knockdown by selective siRNA prevented the synergy between capsaicin and docetaxel (Fig. 4b). These results indicate that targeting AMPK impairs the antiproliferative effect of the combination of DTX and CAP suggesting a role for AMPK in the CAP-induced sensitization to DTX.

### Capsaicin inhibits Akt independently of AMPK activation

**Fig. 5:**
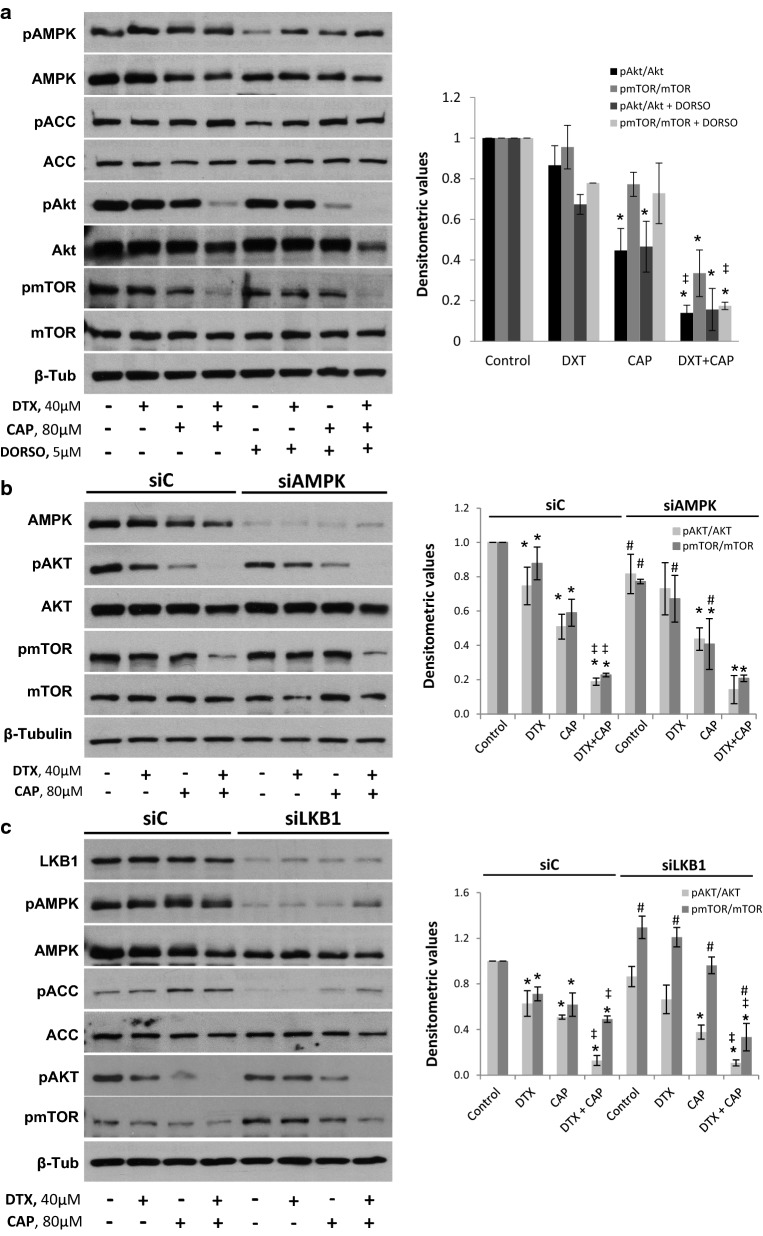
Akt/mTOR inhibition by capsaicin is independent of AMPK activation. Effect of the AMPK inhibitor dorsomorphin (DORSO, 5μM), or AMPK silencing by siRNA or LKB1 silencing by siRNA on Akt/mTOR signaling pathway. **a** PC3 cells were incubated with 40 μM docetaxel (DTX), 80 μM capsaicin (CAP) or the combination of both in the presence or not of 5 μM Dorsomorphin (DORSO) during 1 h. **b** PC3 cells were transfected with sicontrol (siC) or selective siAMPK for 72 h and then treated with 40 μM docetaxel (DTX), 80 μM capsaicin (CAP) or the combination of both during 1 h. **c** PC3 cells were transfected with sicontrol or selective siLKB1 for 72 h and then treated with 40 μM docetaxel (DTX), 80 μM capsaicin (CAP) or the combination of both during 1 h. Levels of proteins were determined by Western blot. β-Tubulin serves as a loading control. The densitometric analyses of bands represented as the mean ± SD of three different experiments are shown on the right. *p < 0.05 significant difference between treated and control cells by two-way ANOVA and Dunnett’s multiple comparisons test; ^#^p < 0.05 significant difference between non-silenced and silenced cells by two-way ANOVA and Dunnett’s multiple comparisons test; ^‡^p < 0.05 indicates significant interaction between DTX and CAP treatment

Recent studies show that AMPK and Akt display antagonistic roles in cellular homeostasis. Furthermore, AMPK and AKT can phosphorylate each other modulating their respective activities. To explore the cross-talk between AMPK and Akt in PC3 cells, AMPK was either pharmacologically inhibited with DORSO or genetically silenced by siRNA, and Akt phosphorylation was determined by Western blotting. As shown in Fig. 5, AMPK targeting by DORSO or by interfering siRNA, did not modify Akt inhibition induced by CAP alone or by the combination of DTX and CAP. Likewise, knocking down LKB1 with siRNA did not prevent CAP-promoted Akt inhibition. Interestingly, mTOR phosphorylation was significantly increased in LKB1 depleted cells (Fig. 5c) according to the well-established inhibition of mTOR by the LKB1/AMPK axe independently of Akt [20, 21]. These results indicate that AMPK activation is not the only responsible for Akt inhibition since appreciable effect of capsaicin on Akt inhibition could be still seen in AMPK-depleted cells, suggesting independent pathways.

### In vivo anti-tumor study

**Fig. 6:**
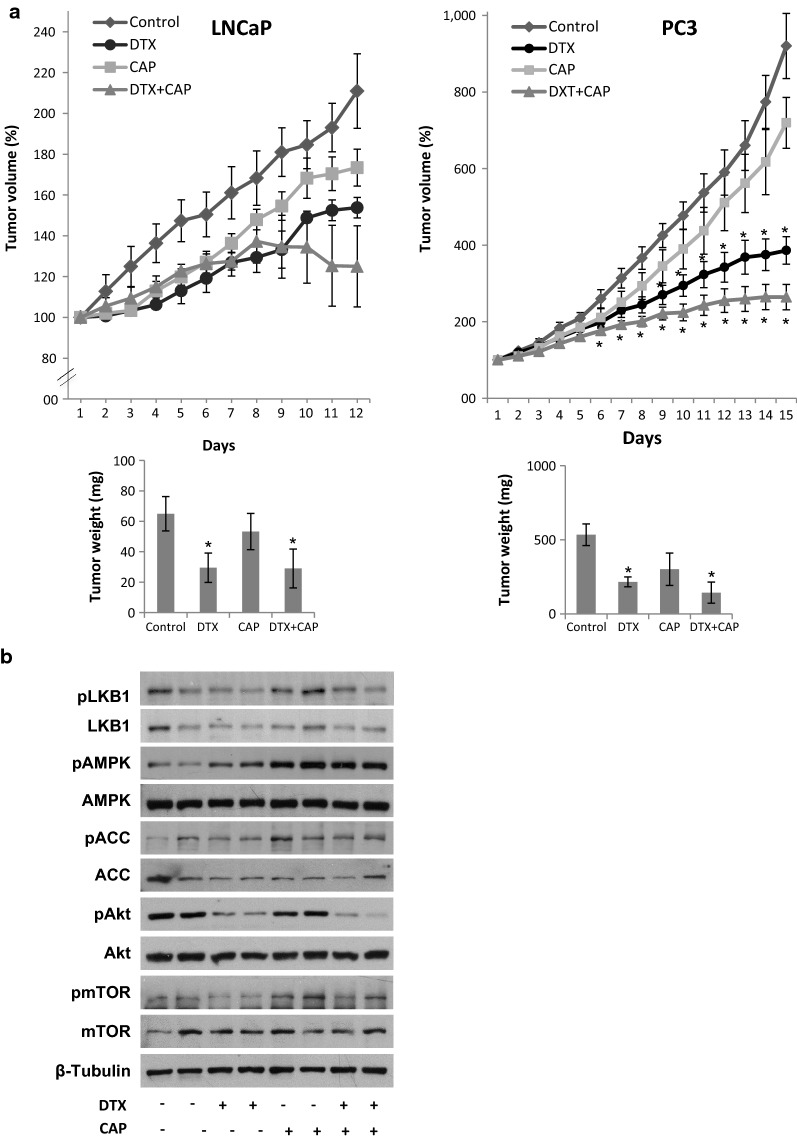
In vivo anti-tumor activity of Docetaxel and Capsaicin combination. Athymic nude mice were injected s.c. in the right flank with LNCaP or PC3 cells. When tumors reached a 70 mm^3^ size, mice were daily treated by i.p. injection with vehicle (control), 10 mg/kg Docetaxel (DTX), 2 mg/kg Capsaicin (CAP) or both compounds (DTX + CAP). Tumor volumes were measured daily. **a** LNCaP tumor growth curve (left) or PC3 tumor growth curve (right) after administration of vehicle (diamonds), DTX (circles), CAP (squares) or both compounds (triangles). Results represent the mean ± SEM of six mice in each group. *p < 0.05 significant difference between treated and control mice by two-way ANOVA and Tukey’s multiple comparisons test. Below is shown the tumor weight at the end of the treatment. **b** Immunoblot analysis of the regulatory signalling pathways in the PC3 dissected tumors

To evaluate the therapeutic efficacy of the combination of docetaxel and capsaicin in vivo, xenograft LNCaP and PC3 tumors were induced in nude mice by s.c. flank injection. When tumors reached 70 mm^3^ volume, the animals were assigned randomly to various groups (n = 6) and injected intraperitoneally with capsaicin or docetaxel alone or with the combination of both compounds. LNCaP tumors growth very slowly according to the less aggressive behavior of this androgen-sensitive cell line (Fig. 6a). Treatment of LNCaP tumors with DTX or CAP singly or in combination moderately reduced tumor growth. In PC3 tumors, docetaxel treatment significantly decreased the growth from day six and DTX + CAP co-treatment could achieve a significantly potent antitumor efficacy (Fig. 6a). It is worthy to note that the combination of docetaxel and capsaicin showed greater antitumor activity than either agent alone and that this effect was synergistic from day 3, as deduced from the combination index (Additional file 1: Fig. S1). By the end of the 15 days treatments, the PC3 tumor volume from mice treated with 2 mg/kg CAP was 76% of the controls, that from mice treated with 10 mg/kg DTX was 55% and the tumor volume from mice co-treated with DTX + CAP was 22% of the controls (Fig. 6a). Likewise, the tumors that were treated with combination therapy had significantly lower wet weight than the tumors in the mice treated with either docetaxel or capsaicin alone (Fig. 6a). No significant change in mice weights was observed during the treatment, indicating that no general toxicity occurred by the treatments (Additional file 2: Table S1  and Additional file 3: Table S2).

Finally, we tested whether AMPK activity was upregulated in the PC3 tumors of nude mice. Figure 6b shows that, in line with the in vitro results, the AMPK activity in the PC3 tumors treated with the combination of CAP and DTX was substantially higher than in those treated with capsaicin alone or docetaxel alone. In addition, Akt phosphorylation was nearly detected in co-treated tumors and was less than in those tumors treated with CAP or DTX singly (Fig. 6b). These results indicate that the combination of capsaicin and docetaxel synergistically reduces PC3 tumor growth in vivo and is very effective for this model of castration resistant prostate cancer.

## Discussion

Docetaxel is considered the most promising anticancer drug for prostate cancer treatment. However, the quick emergence of resistance and systemic toxicity diminished its efficacy. Combination therapy represents a promising therapeutic strategy to overcome toxicity by reduction of the effective dose. Several promising agents are emerging with a potential role in docetaxel-based combinations based on efficacy and manageable toxicity. Preclinical findings suggest that combining such innovative strategies with traditional treatments offers new benefits improving treatment outcome [22, 23]. In this study we evaluated the effectiveness of combining docetaxel and the natural compound capsaicin to reduce prostate tumor growth. We found that the combination of both compounds exhibited synergistic antitumor effect both in vitro and in vivo. Similar results have been previously reported with other compounds used in combination with docetaxel [24–26]. Our results show that the combination of docetaxel and capsaicin caused a strong decrease in the levels of pAkt, pmTOR and pS6 and that targeting this pathway abolishes the cell viability inhibition induced by docetaxel and the synergistic effect.

Recent data indicate that capsaicin displays synergism with diverse conventional drugs as camptothecin [7], pirarubicin [27], brassinin [9] and resveratrol in several tumor cell lines [28]. Nevertheless, the molecular mechanisms involved in this synergistic effect continue to be largely elusive. Our results show that combination of CAP and DTX increases AMPKα catalytic subunit phosphorylation in Thr179 and the phosphorylation of its downstream substrate ACC. Pharmacological inhibition of AMPK as well as AMPK or LKB1 knocking down by siRNA abrogates the capsaicin-dependent inhibition of cell growth and hampers the synergistic effect, indicating that AMPK activation by capsaicin is critical for the antiproliferative effect. Moreover, we propose that the Akt/mTOR axe inhibition by the co-administration was independent of AMPK activation, since AMPK knocking down and inhibition did not have effect on capsaicin-induced Akt downregulation. These results are in agreement with the notion that synergy implies multiple sites of action by definition [29]. Therefore, docetaxel and capsaicin, by regulating two independent pathways, potentiate each other and synergistically inhibit prostate cell viability. In line with our results, it has been shown that combined treatment of the AKT inhibitor perifosine and the AMPK activator AICAR, markedly suppressed prostate PC3 cell growth compared to either treatment alone which indicates that concurrent modulation of AKT and AMPK is more effective than either alone in prostate cancer therapy [30]. Therefore, the co-administration of capsaicin and docetaxel might trigger two signaling pathways that together produce a synergic effect that mediate cancer cell death and growth inhibition.

To further investigate the synergistic antitumor effect of the combination of docetaxel and capsaicin we induced xenograft tumors in nude mice which were treated with CAP, DTX or their combination. According to published data regarding capsaicin bioavailability and absorption [31], for in vivo studies we used a dose of capsaicin equivalent to that used with cells (considering a mice blood volume of 2.5 ml and an average mice weight of 30 g, 80 µM is equivalent to 2 mg/kg). On the other hand, DTX has low bioavailability mainly due to its poor aqueous solubility and its transportation in blood by binding to plasma proteins such as lipoproteins, albumin and α1 acid glycoprotein. Therefore, in vivo doses of docetaxel are usually higher than that used in cells. Thus, we choose a docetaxel dose of 10 mg/kg which is a common used dose in the in vivo studies [32–34]. In LNCaP tumors CAP or DTX singly administered or in combination, had little effect on tumor growth. However, in PC3 tumors, DTX and CAP significantly decreased tumor growth and the DTX + CAP combination had stronger anti-tumor activity that either compound singly administered. Co-treatment induced a robust AMPK activation and Akt/mTOR axe inhibition in the PC3 prostate tumors. Therefore, we demonstrated that the proposed combination of docetaxel and capsaicin potently inhibited the growth of castration resistant prostate cancer cells in vitro and in vivo.

## Conclusion

In conclusion, these findings indicate that docetaxel and capsaicin co-administration represents a therapeutically relevant strategy to improve docetaxel chemotherapy in prostate cancer patients. The fact that AMPK activation is in the underpinning mechanism that sensitizes prostate cells to docetaxel suggests that impact metabolism could be a new option to modulate chemotherapeutic drugs effect.

## Additional files

**Additional file 1: Figure S1.** Isobologram and combination index (CI) of the combined treatment (DTX + CAP) inhibitory effect on LNCaP and PC3 xenograft tumor growth.

**Additional file 2: Table S1.** LNCaP xenografts-wearing mice weights during the treatment.

**Additional file 3: Table S2.** PC3 xenografts-wearing mice weighs during the treatment.

## Abbreviations

ACC: acetyl CoA carboxylase
AMPK: AMP-activated kinase
CI: combination index
CAP: capsaicin
DORSO: dorsomorphin
DTX: docetaxel
LKB1: liver kinase B
