# Supplementary material for: Combination of the natural product capsaicin and docetaxel synergistically kills human prostate cancer cells through the metabolic regulator AMP-activated kinase
Source: Cancer Cell Int. 2019 Mar 8;19:54. doi: 10.1186/s12935-019-0769-2 (PMC6408806; doi:10.1186/s12935-019-0769-2)
Supplement: Supplementary file 1 — Additional file 1: Figure S1. Isobologram and combination index (CI) of the combined treatment (DTX + CAP) inhibitory effect on LNCaP and PC3 xenograft tumor growth. [file 12935_2019_769_MOESM1_ESM.pptx]

## Slide 1
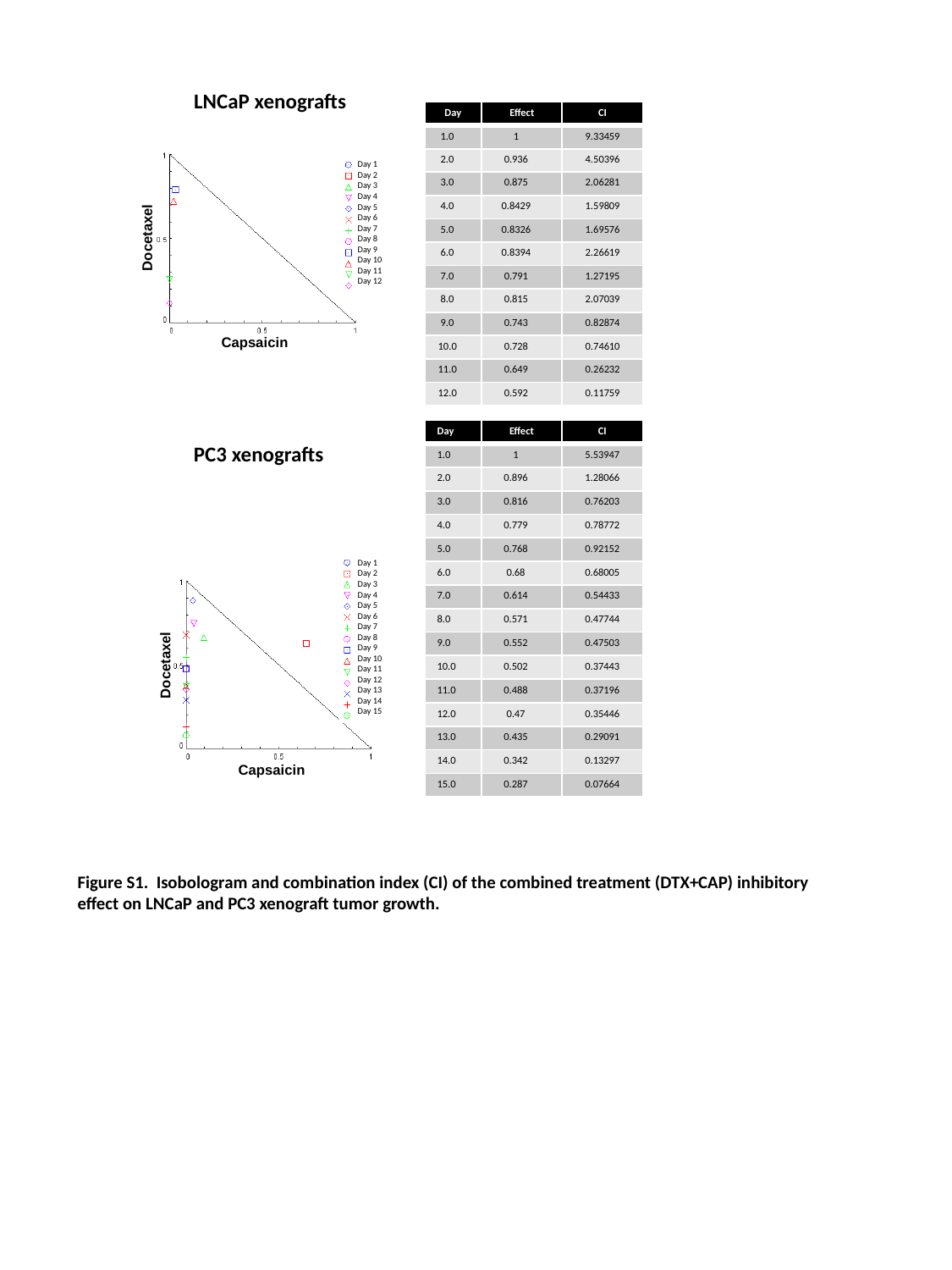

LNCaP xenografts
| Day | Effect | CI |
| --- | --- | --- |
| 1.0 | 1 | 9.33459 |
| 2.0 | 0.936 | 4.50396 |
| 3.0 | 0.875 | 2.06281 |
| 4.0 | 0.8429 | 1.59809 |
| 5.0 | 0.8326 | 1.69576 |
| 6.0 | 0.8394 | 2.26619 |
| 7.0 | 0.791 | 1.27195 |
| 8.0 | 0.815 | 2.07039 |
| 9.0 | 0.743 | 0.82874 |
| 10.0 | 0.728 | 0.74610 |
| 11.0 | 0.649 | 0.26232 |
| 12.0 | 0.592 | 0.11759 |
Docetaxel
Capsaicin
Day 1
Day 2
Day 3
Day 4
Day 5
Day 6
Day 7
Day 8
Day 9
Day 10
Day 11
Day 12
| Day | Effect | CI |
| --- | --- | --- |
| 1.0 | 1 | 5.53947 |
| 2.0 | 0.896 | 1.28066 |
| 3.0 | 0.816 | 0.76203 |
| 4.0 | 0.779 | 0.78772 |
| 5.0 | 0.768 | 0.92152 |
| 6.0 | 0.68 | 0.68005 |
| 7.0 | 0.614 | 0.54433 |
| 8.0 | 0.571 | 0.47744 |
| 9.0 | 0.552 | 0.47503 |
| 10.0 | 0.502 | 0.37443 |
| 11.0 | 0.488 | 0.37196 |
| 12.0 | 0.47 | 0.35446 |
| 13.0 | 0.435 | 0.29091 |
| 14.0 | 0.342 | 0.13297 |
| 15.0 | 0.287 | 0.07664 |
PC3 xenografts
Day 1
Day 2
Day 3
Day 4
Day 5
Day 6
Day 7
Day 8
Day 9
Day 10
Day 11
Day 12
Day 13
Day 14
Day 15
Docetaxel
Capsaicin
Figure S1. Isobologram and combination index (CI) of the combined treatment (DTX+CAP) inhibitory effect on LNCaP and PC3 xenograft tumor growth.
