# Supplementary material for: Combination of the natural product capsaicin and docetaxel synergistically kills human prostate cancer cells through the metabolic regulator AMP-activated kinase
Source: Cancer Cell Int. 2019 Mar 8;19:54. doi: 10.1186/s12935-019-0769-2 (PMC6408806; doi:10.1186/s12935-019-0769-2)
Supplement: Supplementary file 2 — Additional file 2: Table S1. LNCaP xenografts-wearing mice weights during the treatment. [file 12935_2019_769_MOESM2_ESM.pptx]

## Slide 1
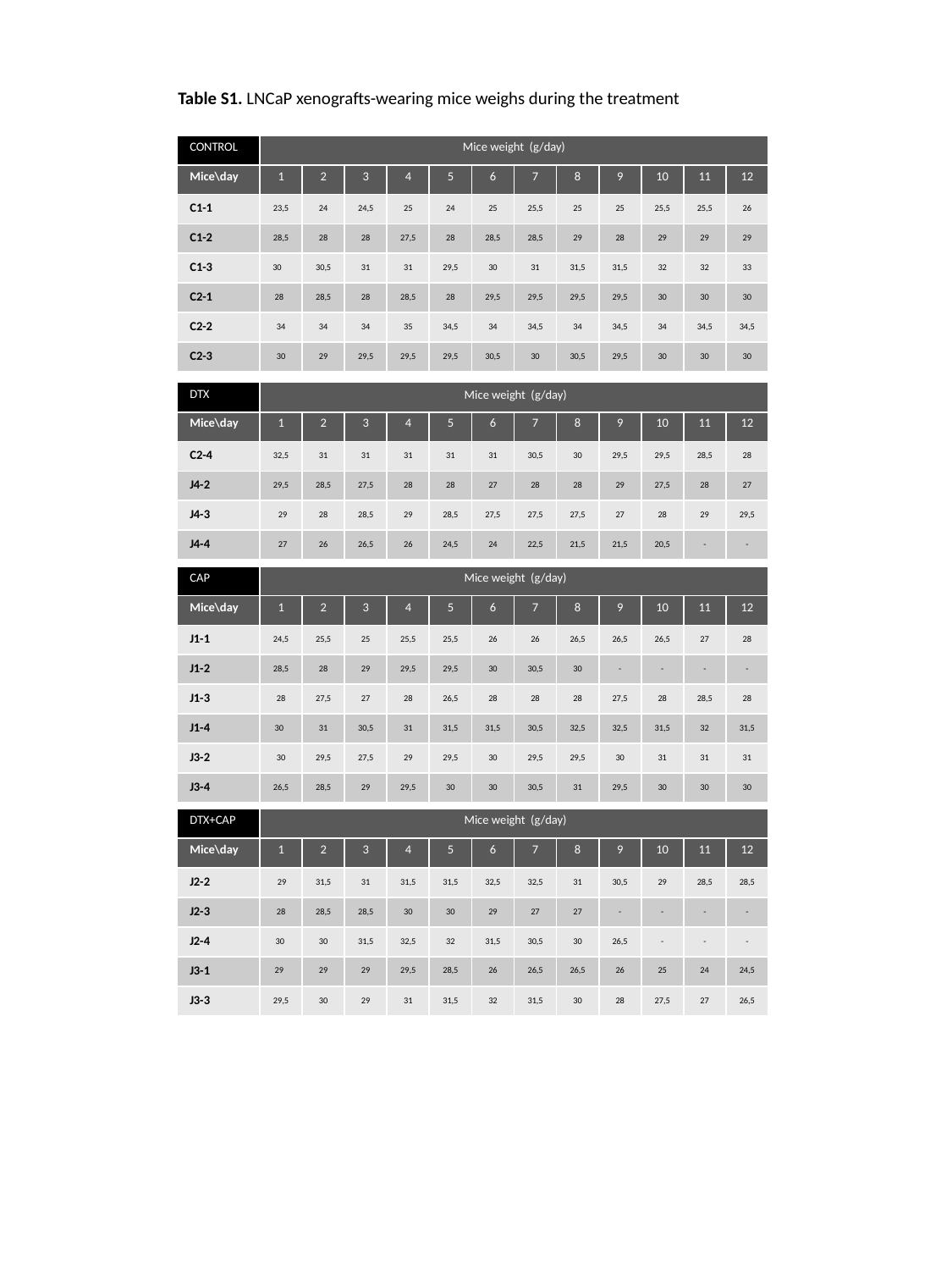

Table S1. LNCaP xenografts-wearing mice weighs during the treatment
| CONTROL | Mice weight (g/day) | | | | | | | | | | | |
| --- | --- | --- | --- | --- | --- | --- | --- | --- | --- | --- | --- | --- |
| Mice\day | 1 | 2 | 3 | 4 | 5 | 6 | 7 | 8 | 9 | 10 | 11 | 12 |
| C1-1 | 23,5 | 24 | 24,5 | 25 | 24 | 25 | 25,5 | 25 | 25 | 25,5 | 25,5 | 26 |
| C1-2 | 28,5 | 28 | 28 | 27,5 | 28 | 28,5 | 28,5 | 29 | 28 | 29 | 29 | 29 |
| C1-3 | 30 | 30,5 | 31 | 31 | 29,5 | 30 | 31 | 31,5 | 31,5 | 32 | 32 | 33 |
| C2-1 | 28 | 28,5 | 28 | 28,5 | 28 | 29,5 | 29,5 | 29,5 | 29,5 | 30 | 30 | 30 |
| C2-2 | 34 | 34 | 34 | 35 | 34,5 | 34 | 34,5 | 34 | 34,5 | 34 | 34,5 | 34,5 |
| C2-3 | 30 | 29 | 29,5 | 29,5 | 29,5 | 30,5 | 30 | 30,5 | 29,5 | 30 | 30 | 30 |
| DTX | Mice weight (g/day) | | | | | | | | | | | |
| --- | --- | --- | --- | --- | --- | --- | --- | --- | --- | --- | --- | --- |
| Mice\day | 1 | 2 | 3 | 4 | 5 | 6 | 7 | 8 | 9 | 10 | 11 | 12 |
| C2-4 | 32,5 | 31 | 31 | 31 | 31 | 31 | 30,5 | 30 | 29,5 | 29,5 | 28,5 | 28 |
| J4-2 | 29,5 | 28,5 | 27,5 | 28 | 28 | 27 | 28 | 28 | 29 | 27,5 | 28 | 27 |
| J4-3 | 29 | 28 | 28,5 | 29 | 28,5 | 27,5 | 27,5 | 27,5 | 27 | 28 | 29 | 29,5 |
| J4-4 | 27 | 26 | 26,5 | 26 | 24,5 | 24 | 22,5 | 21,5 | 21,5 | 20,5 | - | - |
| CAP | Mice weight (g/day) | | | | | | | | | | | |
| --- | --- | --- | --- | --- | --- | --- | --- | --- | --- | --- | --- | --- |
| Mice\day | 1 | 2 | 3 | 4 | 5 | 6 | 7 | 8 | 9 | 10 | 11 | 12 |
| J1-1 | 24,5 | 25,5 | 25 | 25,5 | 25,5 | 26 | 26 | 26,5 | 26,5 | 26,5 | 27 | 28 |
| J1-2 | 28,5 | 28 | 29 | 29,5 | 29,5 | 30 | 30,5 | 30 | - | - | - | - |
| J1-3 | 28 | 27,5 | 27 | 28 | 26,5 | 28 | 28 | 28 | 27,5 | 28 | 28,5 | 28 |
| J1-4 | 30 | 31 | 30,5 | 31 | 31,5 | 31,5 | 30,5 | 32,5 | 32,5 | 31,5 | 32 | 31,5 |
| J3-2 | 30 | 29,5 | 27,5 | 29 | 29,5 | 30 | 29,5 | 29,5 | 30 | 31 | 31 | 31 |
| J3-4 | 26,5 | 28,5 | 29 | 29,5 | 30 | 30 | 30,5 | 31 | 29,5 | 30 | 30 | 30 |
| DTX+CAP | Mice weight (g/day) | | | | | | | | | | | |
| --- | --- | --- | --- | --- | --- | --- | --- | --- | --- | --- | --- | --- |
| Mice\day | 1 | 2 | 3 | 4 | 5 | 6 | 7 | 8 | 9 | 10 | 11 | 12 |
| J2-2 | 29 | 31,5 | 31 | 31,5 | 31,5 | 32,5 | 32,5 | 31 | 30,5 | 29 | 28,5 | 28,5 |
| J2-3 | 28 | 28,5 | 28,5 | 30 | 30 | 29 | 27 | 27 | - | - | - | - |
| J2-4 | 30 | 30 | 31,5 | 32,5 | 32 | 31,5 | 30,5 | 30 | 26,5 | - | - | - |
| J3-1 | 29 | 29 | 29 | 29,5 | 28,5 | 26 | 26,5 | 26,5 | 26 | 25 | 24 | 24,5 |
| J3-3 | 29,5 | 30 | 29 | 31 | 31,5 | 32 | 31,5 | 30 | 28 | 27,5 | 27 | 26,5 |
