# Supplementary material for: Combination of the natural product capsaicin and docetaxel synergistically kills human prostate cancer cells through the metabolic regulator AMP-activated kinase
Source: Cancer Cell Int. 2019 Mar 8;19:54. doi: 10.1186/s12935-019-0769-2 (PMC6408806; doi:10.1186/s12935-019-0769-2)
Supplement: Supplementary file 3 — Additional file 3: Table S2. PC3 xenografts-wearing mice weighs during the treatment. [file 12935_2019_769_MOESM3_ESM.pptx]

## Slide 1
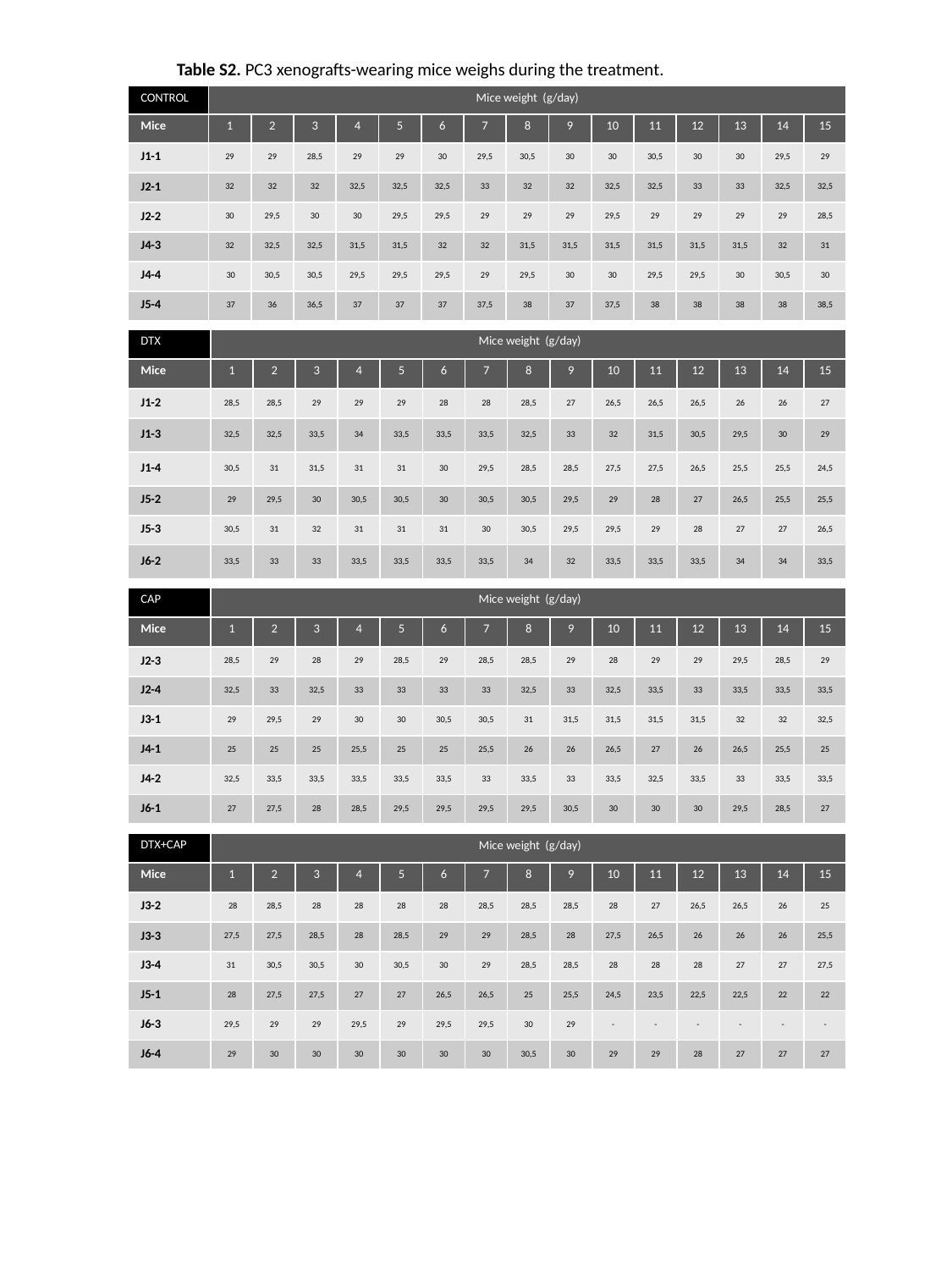

Table S2. PC3 xenografts-wearing mice weighs during the treatment.
| CONTROL | Mice weight (g/day) | | | | | | | | | | | | | | |
| --- | --- | --- | --- | --- | --- | --- | --- | --- | --- | --- | --- | --- | --- | --- | --- |
| Mice | 1 | 2 | 3 | 4 | 5 | 6 | 7 | 8 | 9 | 10 | 11 | 12 | 13 | 14 | 15 |
| J1-1 | 29 | 29 | 28,5 | 29 | 29 | 30 | 29,5 | 30,5 | 30 | 30 | 30,5 | 30 | 30 | 29,5 | 29 |
| J2-1 | 32 | 32 | 32 | 32,5 | 32,5 | 32,5 | 33 | 32 | 32 | 32,5 | 32,5 | 33 | 33 | 32,5 | 32,5 |
| J2-2 | 30 | 29,5 | 30 | 30 | 29,5 | 29,5 | 29 | 29 | 29 | 29,5 | 29 | 29 | 29 | 29 | 28,5 |
| J4-3 | 32 | 32,5 | 32,5 | 31,5 | 31,5 | 32 | 32 | 31,5 | 31,5 | 31,5 | 31,5 | 31,5 | 31,5 | 32 | 31 |
| J4-4 | 30 | 30,5 | 30,5 | 29,5 | 29,5 | 29,5 | 29 | 29,5 | 30 | 30 | 29,5 | 29,5 | 30 | 30,5 | 30 |
| J5-4 | 37 | 36 | 36,5 | 37 | 37 | 37 | 37,5 | 38 | 37 | 37,5 | 38 | 38 | 38 | 38 | 38,5 |
| DTX | Mice weight (g/day) | | | | | | | | | | | | | | |
| --- | --- | --- | --- | --- | --- | --- | --- | --- | --- | --- | --- | --- | --- | --- | --- |
| Mice | 1 | 2 | 3 | 4 | 5 | 6 | 7 | 8 | 9 | 10 | 11 | 12 | 13 | 14 | 15 |
| J1-2 | 28,5 | 28,5 | 29 | 29 | 29 | 28 | 28 | 28,5 | 27 | 26,5 | 26,5 | 26,5 | 26 | 26 | 27 |
| J1-3 | 32,5 | 32,5 | 33,5 | 34 | 33,5 | 33,5 | 33,5 | 32,5 | 33 | 32 | 31,5 | 30,5 | 29,5 | 30 | 29 |
| J1-4 | 30,5 | 31 | 31,5 | 31 | 31 | 30 | 29,5 | 28,5 | 28,5 | 27,5 | 27,5 | 26,5 | 25,5 | 25,5 | 24,5 |
| J5-2 | 29 | 29,5 | 30 | 30,5 | 30,5 | 30 | 30,5 | 30,5 | 29,5 | 29 | 28 | 27 | 26,5 | 25,5 | 25,5 |
| J5-3 | 30,5 | 31 | 32 | 31 | 31 | 31 | 30 | 30,5 | 29,5 | 29,5 | 29 | 28 | 27 | 27 | 26,5 |
| J6-2 | 33,5 | 33 | 33 | 33,5 | 33,5 | 33,5 | 33,5 | 34 | 32 | 33,5 | 33,5 | 33,5 | 34 | 34 | 33,5 |
| CAP | Mice weight (g/day) | | | | | | | | | | | | | | |
| --- | --- | --- | --- | --- | --- | --- | --- | --- | --- | --- | --- | --- | --- | --- | --- |
| Mice | 1 | 2 | 3 | 4 | 5 | 6 | 7 | 8 | 9 | 10 | 11 | 12 | 13 | 14 | 15 |
| J2-3 | 28,5 | 29 | 28 | 29 | 28,5 | 29 | 28,5 | 28,5 | 29 | 28 | 29 | 29 | 29,5 | 28,5 | 29 |
| J2-4 | 32,5 | 33 | 32,5 | 33 | 33 | 33 | 33 | 32,5 | 33 | 32,5 | 33,5 | 33 | 33,5 | 33,5 | 33,5 |
| J3-1 | 29 | 29,5 | 29 | 30 | 30 | 30,5 | 30,5 | 31 | 31,5 | 31,5 | 31,5 | 31,5 | 32 | 32 | 32,5 |
| J4-1 | 25 | 25 | 25 | 25,5 | 25 | 25 | 25,5 | 26 | 26 | 26,5 | 27 | 26 | 26,5 | 25,5 | 25 |
| J4-2 | 32,5 | 33,5 | 33,5 | 33,5 | 33,5 | 33,5 | 33 | 33,5 | 33 | 33,5 | 32,5 | 33,5 | 33 | 33,5 | 33,5 |
| J6-1 | 27 | 27,5 | 28 | 28,5 | 29,5 | 29,5 | 29,5 | 29,5 | 30,5 | 30 | 30 | 30 | 29,5 | 28,5 | 27 |
| DTX+CAP | Mice weight (g/day) | | | | | | | | | | | | | | |
| --- | --- | --- | --- | --- | --- | --- | --- | --- | --- | --- | --- | --- | --- | --- | --- |
| Mice | 1 | 2 | 3 | 4 | 5 | 6 | 7 | 8 | 9 | 10 | 11 | 12 | 13 | 14 | 15 |
| J3-2 | 28 | 28,5 | 28 | 28 | 28 | 28 | 28,5 | 28,5 | 28,5 | 28 | 27 | 26,5 | 26,5 | 26 | 25 |
| J3-3 | 27,5 | 27,5 | 28,5 | 28 | 28,5 | 29 | 29 | 28,5 | 28 | 27,5 | 26,5 | 26 | 26 | 26 | 25,5 |
| J3-4 | 31 | 30,5 | 30,5 | 30 | 30,5 | 30 | 29 | 28,5 | 28,5 | 28 | 28 | 28 | 27 | 27 | 27,5 |
| J5-1 | 28 | 27,5 | 27,5 | 27 | 27 | 26,5 | 26,5 | 25 | 25,5 | 24,5 | 23,5 | 22,5 | 22,5 | 22 | 22 |
| J6-3 | 29,5 | 29 | 29 | 29,5 | 29 | 29,5 | 29,5 | 30 | 29 | - | - | - | - | - | - |
| J6-4 | 29 | 30 | 30 | 30 | 30 | 30 | 30 | 30,5 | 30 | 29 | 29 | 28 | 27 | 27 | 27 |
